# Self-Efficacy and Psychological Well-Being in Adolescents: Evaluating the Moderating Role of Socioeconomic Status and Cultural Factors

**DOI:** 10.3390/pediatric17020039

**Published:** 2025-03-21

**Authors:** Giulia Raimondi, James Dawe, Fabio Alivernini, Sara Manganelli, Pierluigi Diotaiuti, Laura Mandolesi, Michele Zacchilli, Fabio Lucidi, Elisa Cavicchiolo

**Affiliations:** 1 Department of Developmental and Social Psychology, Sapienza University of Rome, Via dei Marsi 78, 00185 Rome, Italy; 2 Department of Human, Social and Health Sciences, University of Cassino and Southern Lazio, Via S. Angelo, Campus Folcara, 03043 Cassino, Italy; 3 Department of Humanities, Federico II University of Naples, Corso Umberto I 40, 80138 Naples, Italy; 4 Department of Systems Medicine, Tor Vergata University of Rome, Via Montpellier 1, 00133 Rome, Italy

**Keywords:** academic self-efficacy, moderation analysis, socioeconomic status, immigrant background, emotional well-being

## Abstract

Background/Objectives: Adolescence is a crucial developmental stage characterized by significant psychological and emotional changes. Within the school context, academic self-efficacy (ASE) influences students’ emotional well-being, including positive and negative affective states. Research has shown that both ASE and emotional well-being are associated with socioeconomic status (SES) and immigrant background. This study aims to examine whether SES and immigrant background moderate the relationship between ASE and positive/negative affect among adolescents. Methods: Data were collected from a representative sample of 26,564 10th-grade students in Italian schools. ASE, positive and negative affect, SES, and immigrant background were assessed through validated measures. Multigroup structural equation modeling (multigroup SEM) was conducted to test the moderating roles of SES (low, middle, high) and immigrant background (native, first-generation immigrant, second-generation immigrant) on the relationship between ASE and affective states. Results: The results indicated that ASE significantly predicted positive affect (β = 0.34, *p* < 0.001) and negative affect (β = −0.17, *p* < 0.001) across all groups. However, results indicated no significant differences in the ASE–emotional affect relationship across SES and immigrant background groups, indicating that neither SES nor immigrant background moderated these associations. Conclusions: The findings suggest that ASE is associated with both positive affect and negative affect in adolescents, irrespective of SES and immigrant background. This highlights the universal importance of fostering ASE in school settings to support emotional well-being across diverse demographic groups.

## 1. Introduction

Adolescence is a crucial developmental period during which individuals experience profound changes in their psychological, emotional, and social functioning [1]. Since adolescents spend most of their time in school, it is of utmost importance to study their well-being within the school context. Among the factors that contribute to adolescent students’ overall well-being, academic self-efficacy has been extensively studied as a core construct influencing not only academic outcomes but also emotional regulation and psychological health [2]. Academic self-efficacy (ASE), as defined by Bandura [3], refers to an individual’s belief in their ability to successfully carry out tasks and achieve goals. High ASE is associated with greater engagement in adaptive behaviors, setting challenging goals, and persisting through difficulties, fostering resilience and positive emotional experiences in adolescents [4,5]. Conversely, low ASE is often associated with avoidance of challenges, increased academic stress, and the development of negative emotional states such as anxiety, frustration, and helplessness [6].

While other constructs (e.g., motivation, emotion regulation, peer relationships) have been shown to influence adolescents’ well-being as well [7], ASE is a psychological resource directly associated with students’ ability to regulate their learning behaviors and cope with academic challenges. Compared to broader constructs, such as self-esteem or emotion regulation, ASE offers a more context-specific measure of how students perceive their competence in handling school-related tasks, which has immediate implications for their emotional experiences within the school context [8].

### 1.1. Theoretical Background

The impact of ASE extends to various facets of psychological well-being, particularly in relation to affective states. Affective states, including both positive emotions (e.g., happy and calm) and negative emotions (e.g., sad and upset), are key indicators of an individual’s psychological health [9]. Research has shown that adolescents with higher levels of ASE tend to experience more positive emotions, which can buffer against the development of mental health issues [5,10]. On the other hand, adolescents with lower ASE are more likely to experience negative emotions, which can undermine their psychological well-being and academic success [2]. However, there might be factors related to students’ background characteristics that play a crucial role in moderating the relationship between self-efficacy and its influence on affective states. For example, research has demonstrated that factors such as socioeconomic status (SES) and immigrant background play a significant role in shaping adolescents’ experiences of ASE [11,12,13,14]. SES is usually measured as a combination of different factors (e.g., family income, educational level, and occupational status), and it is known to affect educational outcomes through low access to educational resources and opportunities [15]. In fact, previous studies found that students from lower SES backgrounds reported significantly lower levels of ASE compared to their higher SES peers, which could be due to difficulties in access to academic resources, supportive networks, and environments that foster self-confidence [13,14,16]. Lower SES students may also face additional stressors, such as financial instability and limited opportunities for academic engagement, which undermine their ability to develop strong self-efficacy beliefs [17]. By contrast, students from higher SES backgrounds benefit from enriched academic contexts, including access to extracurricular opportunities and more supportive parental involvement, which enhance their ASE [17,18]. Similarly, the immigrant background is another crucial factor that can affect ASE. In recent years, the movements of people across countries’ borders have become more complex and frequent, which could have an impact on educational opportunities and systems [19]. Adolescents with an immigrant background often face unique challenges, including language barriers, cultural adaptation issues, and feelings of exclusion, which can negatively impact their self-efficacy in academic contexts [11,20,21], leading to higher dropout rates as well [22]. These barriers may create additional challenges for developing the competence needed to engage successfully in academic tasks. However, research also indicates that acculturation processes, such as familiarity with the host country’s language and cultural norms, can mitigate these challenges, fostering higher ASE levels among immigrant students who experience greater social and educational integration [23,24].

Therefore, understanding the interplay between ASE and affective states in light of these possible moderators is important for promoting adolescents’ psychological health and fostering environments that support positive development.

### 1.2. Positive and Negative Affect and the Relationships with Socioeconomic Status and Immigrant Background

Research has shown that individuals from lower socioeconomic backgrounds often experience heightened emotional sensitivity to negative emotional stimuli, compared to individuals from higher socioeconomic backgrounds, who, in turn, tend to respond more strongly to positive emotional stimuli [25,26]. These differences have been attributed to chronic exposure to stressors, which are more prevalent in low-SES environments, such as financial instability, neighborhood dangers, and social difficulties, and can create a persistent state of vigilance, making individuals more sensitive to challenges [25,27]. Conversely, individuals from higher SES backgrounds tend to have greater access to supportive environments and resources that foster resilience and more positive emotional processing [27,28]. Therefore, in the educational context, this heightened sensitivity to stress can lead adolescents from lower SES backgrounds to experience stronger emotional reactions to academic demands [29,30]. Conversely, students from higher SES backgrounds tend to experience more positive affect [28,31], highlighting the role of socioeconomic factors in shaping emotional experiences within the educational context.

Adolescents with an immigrant background often face unique emotional challenges within school and social contexts. These experiences are influenced by how they perceive their surroundings and, crucially, by how they are perceived by others, especially in terms of social acceptance, warmth, and competence [32,33]. Native adolescents rarely encounter stereotypes that question their competence or belonging, allowing them to engage in school activities with a sense of security and inclusion. In contrast, first-generation immigrant adolescents—those born outside the host country—are often confronted with both cultural and language differences, and they are also more likely to be portrayed as less competent [34]. Feeling misunderstood or isolated can heighten their sensitivity to negative emotional experiences, such as rejection, and discomfort [35,36], which can affect their overall academic performance [37,38]. Second-generation immigrant adolescents, born in the host country to immigrant parents, occupy a unique position between cultural identities [32]. While they are more familiar with the cultural norms and values of their surroundings than their first-generation peers, they may still face subtle biases linked to their family’s heritage. This blend of familiarity and perceived difference often leads to mixed emotional experiences, as they may feel only partly accepted by native peers, resulting in emotional conflicts [32].

### 1.3. The Present Study

Despite extensive research on the relationship between self-efficacy and psychological well-being, as well as the well-established association between socioeconomic and cultural variables—such as SES and immigrant background—and ASE [13,24], no studies have specifically examined whether these factors can moderate the relationship between self-efficacy and affective states in adolescents. Understanding these moderating effects is crucial, as adolescents from disadvantaged socioeconomic backgrounds or with an immigrant background may experience greater emotional difficulties, making it harder for them to benefit from the protective effects of high self-efficacy. The current study aims to fill this gap by examining the moderating role of SES and immigrant background in the relationship between self-efficacy and affective states among adolescents in the school context. Specifically, this study investigates how these socioeconomic and cultural factors influence the association between self-efficacy and positive and negative emotions, in order to provide a more comprehensive understanding of the factors that contribute to adolescents’ psychological well-being.

With regard to this objective, we hypothesized the following:

**H1:**  *Given the relationship between ASE and positive and negative affect within the school context, we expect that adolescents with higher levels of ASE will experience more positive affect and less negative affect.*

**H2:**  *Based on previous studies [25,27,39] highlighting the fact that individuals from lower and higher SES backgrounds report different emotional sensitivity processing, we hypothesize that the effect of ASE on positive affect will be stronger for adolescents with a higher SES background. On the other hand, we hypothesize that the effect of ASE on negative affect will be greater for adolescents with a lower SES background.*

**H3:**  *Based on previous studies [32,33], which highlight the unique emotional challenges faced by adolescents with an immigrant background, specifically by first-generation immigrant adolescents, we hypothesize that the effect of ASE on positive affect will be stronger for native adolescents. Conversely, we hypothesize that the effect of ASE on negative affect will be greater for adolescents with an immigrant background, specifically for first-generation immigrant adolescents.*

## 2. Materials and Methods

### 2.1. Participants and Procedure

The current research included data from a representative sample of 26,564 10th-grade students from the Italian population attending upper secondary schools, who participated in the National Evaluation of Learning [40]. The mean age of the students was 15.60 years (SD = 0.76). Among these students, 50.1% were females, 6.6% were first-generation immigrants (i.e., born abroad and with foreign-born parents), and 5.6% were second-generation immigrants (i.e., born in Italy but with both parents born abroad). Each school obtained informed consent and parental permission according to the assessment protocol of the National Evaluation of Learning [40].

### 2.2. Measures

*Academic Self-Efficacy*. A measure of ASE for self-regulated learning was used in order to assess students’ confidence in their skills in time management, taking notes in class, and planning school work, which are crucial for academic success, independent of specific subjects, and needed across different school environments. The items used were as follows: (1)“*Finish homework assignments by deadlines?*”; (2) “*Study when there are other interesting things to do?*”; (3) “*Concentrate on school subjects?*”; (4) “*Remember information presented in class?*” [41]. Since this study was part of a larger project that also included younger students, these items were selected from the original scale to ensure suitability for different school levels. Furthermore, previous studies [42,43] supported the good psychometric properties of the brief scale. Students rated their perceived capability to manage each learning task on a 5-point scale, ranging from 1 (“*Cannot do at all*”) to 5 (“*Highly certain can do*”). The original scale has been used in the Italian context, reporting good psychometric properties [4,44,45]. The Cronbach’s alpha in the current sample was 0.72.

*Immigrant background*. Immigrant background was defined in accordance with the classification of the Organization for Economic Co-operation and Development [46]; (i) native adolescents were defined as being born in Italy and having at least one parent who was born in Italy, (ii) first-generation immigrants were defined as being foreign-born and having parents born abroad, and (iii) second-generation immigrants were defined as being born in Italy and having parents born abroad.

*SES*. Students’ SES [46] was obtained by calculating factor scores from a principal component analysis based on four indicators: (i) the occupational level of parents, (ii) the educational level of parents, (iii) home possessions, and (iv) home literacy resources. Finally, tertiles of the SES scores were calculated in order to categorize students into three groups: lower SES (i.e., SES scores within the first tertile), middle SES (i.e., SES scores within the second tertile), and higher SES (i.e., SES scores within the third tertile).

*Psychological Well-being*. The Feelings at School Scale (FASS) [26] was used to measure students’ positive and negative affect in the school context (i.e., “*If you think about how you felt at school over the past few months, how often did you experience the following feelings?*”). The FASS is composed of 8 items (4 measuring positive affect, including “happy, cheerful, good, calm”, and 4 measuring negative affect, including “sad, upset, worried, angry”), rated on a 5-point scale from 1 (“Never”) to 5 (“Very often”), with satisfactory psychometric properties. The Cronbach’s alphas for the positive affect and negative affect scales in the current sample were 0.84 and 0.72, respectively.

### 2.3. Statistical Analyses

Firstly, confirmatory factor analyses (CFAs) were conducted in order to evaluate the construct validity of ASE and positive/negative affect, ensuring that the measurement models provided an adequate fit to the data. To address whether ASE is associated with positive and negative affect, structural equation modeling (SEM) was initially conducted on the overall sample. In the SEM, ASE was specified as an exogenous latent variable (predictor), while positive and negative affect were specified as endogenous latent variables (outcomes), each defined by their respective observed indicators. Subsequently, multigroup SEM analyses were conducted to assess the moderating role of SES and immigrant background. Specifically, these analyses allowed us to examine whether the strength of the relationships between ASE and positive/negative affect differed significantly across groups based on SES and immigrant background.

The following indices were used to assess model fit: (1) the chi-square (χ^2^) test, with the corrected chi-square difference test (since the MLR estimation was used in the analysis) [47]. However, chi-square is sensitive to sample size because larger samples make even small differences between the observed and expected covariance matrices more likely to be detected. This can result in statistically significant *p*-values that indicate model misfit, even when differences are negligible [48]; (2) the comparative fit index (CFI), with values > 0.95 indicating a good model fit and values of 0.90 and higher an acceptable fit [49]; (3) the Tucker–Lewis index (TLI), with values > 0.95 indicating a good fit of the model and values of 0.90 and higher an acceptable fit [50]; (4) the root mean square error of approximation (RMSEA), with values between 0.05 and 0.08 indicating an adequate fit of the model and values above or equal to 0.10 indicating a poor fit of the model [51,52]; and (5) the standardized root mean square residual (SRMR), with values < 0.08 indicating a good fit [53].

To test for moderation, i.e., whether the effect of ASE on positive and negative affect varies across SES levels (low, middle, high) and immigrant background (native students, first-generation immigrant students, second-generation immigrant students), we compared a model with a freely estimated regression coefficient (β) across groups to a nested model where the β value was constrained to be equal across groups. In both models, the variances of the latent constructs were fixed to be equal across groups, allowing only the regression path to vary in the nested model. The comparison with the nested model was evaluated by using ΔRMSEA (<0.015), ΔCFI (<0.01), and ΔSRMR (<0.01) [54].

All the analyses were performed using Mplus 8.3 [55], with the COMPLEX option to account for the nested structure of the data, and the Statistical Package for Social Sciences [56].

## 3. Results

The CFAs indicated a good fit of each measurement model for ASE (χ^2^ = 297.87, df = 2; RMSEA = 0.07; CFI = 0.98; TLI = 0.95; SRMR = 0.02) and positive/negative affect (χ^2^ = 1769.51, df = 19; RMSEA = 0.06; CFI = 0.97; TLI = 0.95; SRMR = 0.03), supporting the inclusion of these variables as latent dimensions in the following SEM models.

The regression model yielded a good fit (χ^2^ = 2892.26, df = 51; RMSEA = 0.05; CFI = 0.96; TLI = 0.95; SRMR = 0.03) and indicated that ASE is significantly associated with positive and negative affect. Specifically, ASE was positively associated with positive affect (β = 0.34; *p* < 0.001), indicating that higher self-efficacy is linked to increased positive emotions. Conversely, ASE was negatively associated with negative affect (β = −0.17; *p* < 0.001), suggesting that greater self-efficacy is linked to fewer negative emotional experiences.

**Figure 1:**
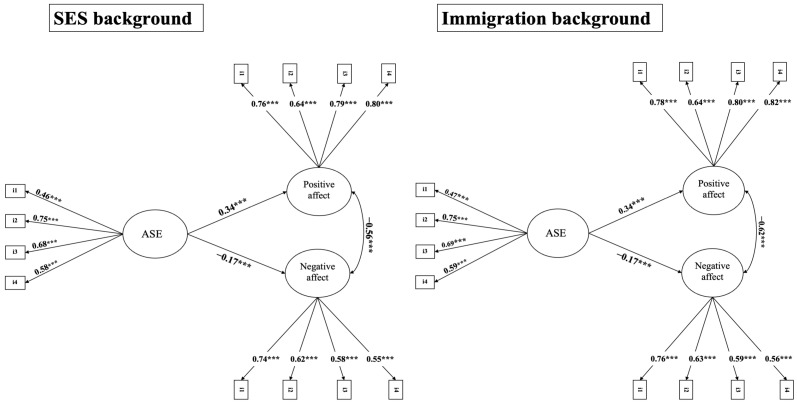
Moderation results for SES and immigrant background. ***Note***. ASE = academic self-efficacy; SES = socioeconomic status. *** *p* < 0.001. Standardized coefficients of the constrained models.

We also tested whether the relationship between ASE and positive and negative affect was moderated by biological sex (i.e., females and males) and regions of Italy (i.e., north, central, and south of Italy). The results indicated that for both these additional moderators, models with the freely estimated regression parameter yielded a good fit to the data (biological sex: χ^2^ = 3777.18, df = 123; RMSEA = 0.05; CFI = 0.95; TLI = 0.95; SRMR = 0.04; regions of Italy: χ^2^ = 3251.03, df = 195; RMSEA = 0.04; CFI = 0.96; TLI = 0.96; SRMR = 0.03), just as the models with the constrained regression parameter (biological sex: χ^2^ = 3799.12, df = 125; RMSEA = 0.05; CFI = 0.95; TLI = 0.95; SRMR = 0.04; regions of Italy: χ^2^ = 3251.73, df = 199; RMSEA = 0.04; CFI = 0.96; TLI = 0.96; SRMR = 0.04). The standardized constrained beta values for biological sex were 0.34 (*p* < 0.001) for positive affect and −0.20 (*p* < 0.001) for negative affect. The standardized constrained beta values for immigrant background were 0.33 (*p* < 0.001) for positive affect and −0.16 (*p* < 0.001) for negative affect. These findings indicated that neither of these two variables moderates the relationship between ASE and positive and negative affect. As regards SES and immigrant background, models with the freely estimated regression parameter yielded a good fit to the data (SES: χ^2^ = 3307.01, df = 195; RMSEA = 0.04; CFI = 0.96; TLI = 0.96; SRMR = 0.03; immigrant background: χ^2^ = 3438.24, df = 195; RMSEA = 0.04; CFI = 0.96; TLI = 0.96; SRMR = 0.03), just like the models with the constrained regression parameter (SES: χ^2^ = 3320.19, df = 199; RMSEA = 0.04; CFI = 0.96; TLI = 0.96; SRMR = 0.04; immigrant background: χ^2^ = 3454.58, df = 199; RMSEA = 0.04; CFI = 0.96; TLI = 0.96; SRMR = 0.04). The model comparison analysis (see Table 1) indicated no differences between the models, suggesting that SES and immigrant background do not moderate the relationship between ASE and positive and negative affect. This result implies that the influence of ASE on positive and negative affect is consistent across SES levels and immigrant background groups (see Figure 1 for the standardized path results of the constrained model and Table 2 for the unconstrained model).

## 4. Discussion

The aim of the current study was to examine the moderating role of socioeconomic status (SES) and immigrant background on the relationship between academic self-efficacy (ASE) and affective states (positive and negative affect) among adolescents within the school context. By investigating the interaction between ASE and these socioeconomic and cultural factors, this study sought to provide a more comprehensive understanding of the factors influencing adolescents’ emotional experiences to foster students’ academic achievement and well-being [10].

In line with our first hypothesis (HP1), our findings confirmed that ASE is a significant predictor of both positive and negative affect in adolescents. Specifically, higher levels of ASE were associated with increased positive affect and decreased negative affect. These results align with the previous literature [5,10] and underscore the role of ASE as a protective factor for adolescents’ psychological well-being. Adolescents with high ASE are likely to approach challenges with a sense of competence and persistence, which fosters positive emotions such as optimism and enthusiasm [2]. Conversely, those with lower ASE may struggle to cope effectively with academic demands, leading to emotional responses like frustration and upset [6]. This result reinforces the role of ASE as an important factor not only for academic achievement but also for supporting positive emotional development during adolescence.

Contrary to our second hypothesis (HP2), SES did not moderate the relationship between ASE and positive or negative affect. Although previous research has suggested that both individuals from lower and higher SES backgrounds may have a differently heightened sensitivity to positive and negative emotional stimuli [25], this heightened emotional reactivity did not appear to affect the impact of ASE on emotional outcomes in our study. One possible explanation for this finding is that the benefits of high ASE, such as resilience and adaptive coping, may function consistently across SES groups, buffering against negative emotions and promoting positive emotions regardless of socioeconomic status. This result suggests that while SES can influence general emotional reactivity [30], its role as a moderator in the ASE–emotional affect relationship may be limited within the structured environment of schools. Another interpretation could be related to the measurement of SES itself [57]. SES is a complex construct encompassing multiple dimensions (e.g., income, education, neighborhood), and the lack of a moderation effect in this study may reflect limitations in capturing the nuanced ways that SES could interact with ASE. In fact, it has been demonstrated that students from a lower SES background have limited access to educational resources (e.g., textbooks, technology, and extracurricular learning opportunities) [58], as well as higher stress levels due to factors outside the educational environment, such as financial instability or familial responsibilities [30,59]. In their study, Conner et al. [60] found a lack of moderation by SES for self-efficacy in different behavioral contexts, such as smoking cessation, breastfeeding, and physical activity. Although the type of self-efficacy assessed in those studies differs from ASE, it is important to note that SES also did not moderate the relationship between self-efficacy and behavioral outcomes. The authors [60] suggested that self-efficacy may act as a stable psychological resource that remains relatively unaffected by socioeconomic disparities. This perspective aligns with our results, reinforcing the idea that self-efficacy plays a protective role across diverse groups, regardless of their SES.

Similarly, our results did not support our third hypothesis, as an immigrant background did not moderate the relationship between ASE and positive or negative affect. This finding is particularly intriguing given existing research indicating that immigrant adolescents, especially first-generation immigrants, often experience unique emotional challenges linked to perceived social acceptance and cultural differences [32,61]. For instance, a recent study [24] found that first-generation immigrant students tend to report lower ASE compared to their native peers, likely due to language barriers and cultural adaptation challenges, which can influence self-efficacy beliefs. While these challenges may strengthen their emotional experiences, the psychological benefits of ASE might remain consistent across different cultural backgrounds, as self-efficacy fosters a sense of control and confidence that may help immigrant adolescents manage emotional challenges despite potential social or cultural obstacles. A possible explanation for the lack of moderation effects observed for SES and immigrant background may lie in the overall school climate and the degree of socioeconomic segregation in schools. According to the OECD report [22] on equity in education, inclusive school policies and a positive learning environment can play a crucial role in promoting resilience and reducing disparities in academic and emotional outcomes for disadvantaged students. The levels of equity (i.e., the degree to which the school system provides learning opportunities to all children) and socioeconomic segregation in schools vary across countries [22]. For example, Italy is among the countries with the highest level of equity and relatively low segregation. This indicates that disadvantaged students in our sample might attend schools with similar resources and opportunities to their more advantaged peers, which could partially mitigate the negative effects of having a low SES or an immigrant background. Moreover, since SES is linked to academic success, which in turn is strongly predicted by ASE and students’ well-being [22,62], the low level of school-based socioeconomic segregation and high level of equity might have helped reduce the expected moderating role of SES in the relationship between ASE and emotional well-being.

The lack of moderating effects for SES and immigrant background highlights the robustness of ASE as a predictor of emotional outcomes. In fact, these findings indicate that the structural relationship between ASE and positive/negative affect holds equally across these groups, suggesting that ASE could be seen as a protective factor, promoting emotional well-being consistently across diverse socioeconomic and cultural contexts. However, our finding also highlights that the school environment itself could play a buffering role by fostering inclusivity and multicultural support, leveling the relationship between ASE and emotional affect across groups.

Finally, even though the standardized coefficients for positive and negative affect indicate moderate effects, they remain relevant in educational contexts, as even moderate effects can lead to meaningful educational outcomes, particularly at a population level. Moreover, such effects can be sustained through targeted support programs in schools, ultimately contributing to the enhancement of adolescents’ emotional well-being.

### 4.1. Limitations and Future Directions

This study is not without limitations. Firstly, although our study was based on a large nationally representative sample, it was focused on 10th-grade students. Further studies are needed to assess whether SES and immigrant background could be moderators of the relationship between ASE and emotional well-being in other school grades. Secondly, although our operationalization of SES was detailed and comprehensive of different features (i.e., measured as (i) the occupational level of parents, (ii) the educational level of parents, (iii) home possessions, and (iv) home literacy resources), it could be interesting to explore SES by differentiating between each individual indicator (e.g., economic hardship, parental education, or neighborhood context), and to examine whether these facets of SES could have distinct moderating effects on ASE and emotional well-being. Thirdly, since the current study is cross-sectional, future research could adopt a longitudinal approach to explore how ASE and emotional well-being evolve over time, providing stronger evidence of causal relationships and offering insights into whether SES and immigrant background influence developmental trajectories. Furthermore, longitudinal data could also help identify critical periods when interventions might be most effective.

Future studies could further explore the role of specific negative emotions, such as indignation and bitterness, which may arise when adolescents experience unmet expectations or feel misunderstood in academic and social contexts. These emotions are particularly relevant in relation to social interactions, identity formation, and perceived injustice [63,64], and might be even more pronounced among adolescents from lower SES backgrounds or those with an immigrant background, who may encounter unique challenges related to social acceptance and cultural integration.

Moreover, it is important to note that while SES and immigrant background did not moderate the relationship between ASE and emotional well-being in the school context, future research could investigate whether these factors play a moderating role in other domains of adolescents’ lives, such as their online experiences. Recent research [65] highlighted the importance of self-efficacy beliefs in helping adolescents navigate digital environments and avoid misinformation, which can have a significant impact on their well-being.

Lastly, future studies could explore other contextual factors, such as school climate or peer relationships, that may affect how adolescents experience and report emotional states. A deeper understanding of these dynamics could help refine intervention strategies and offer more targeted support to students from diverse backgrounds.

### 4.2. Educational Implications

The findings of this study highlight the importance of improving ASE within the school settings, in order to support adolescents’ emotional well-being. Schools could implement targeted interventions aimed at enhancing students’ ASE, for example, through individual mentoring programs to help students set realistic goals and adaptive coping mechanisms. Programs could also focus on teacher training to create supportive classroom environments, as teachers play a crucial role in reinforcing students’ confidence in their abilities. Finally, peer support initiatives could help foster ASE by promoting collaborative learning experiences and fostering mutual support among students, as well as peer acceptance [66].

## 5. Conclusions

The current study contributes to a deeper understanding of the relationship between ASE and emotional well-being in adolescents, showing that these associations hold consistently across diverse socioeconomic and cultural backgrounds. These findings underscore the importance of fostering ASE in school settings as a universal approach to supporting adolescents’ emotional health. Future research should continue to explore the complex interplay of socioeconomic and cultural factors in shaping psychological outcomes, with attention to the potential buffering role of the school environment.

## Figures and Tables

**Table 1:** Model comparison for the moderation analysis.

**SES**											
**Model**	**χ^2^**	**df**	**∆χ^2^**	**∆*df***	**CFI**	**TLI**	**RMSEA**	**SRMR**	**ΔCFI**	**ΔRMSEA**	**ΔSRMR**
Model-1	3307.01	195	-	-	0.958	0.957	0.043	0.034	-	-	-
Model-2	3320.19	199	13.18	4	0.958	0.958	0.042	0.035	0	0.001	0.001
**Immigrant Background**											
**Model**	**χ^2^**	**df**	**∆χ^2^**	**∆*df***	**CFI**	**TLI**	**RMSEA**	**SRMR**	**ΔCFI**	**ΔRMSEA**	**ΔSRMR**
Model-1	3438.24	195	-	-	0.957	0.957	0.044	0.034	-	-	-
Model-2	3454.57	199	16.33	4	0.957	0.957	0.043	0.035	0	0.001	0.001

***Note***. χ^2^ = chi-square; df = degrees of freedom; CFI = comparative fit index; TLI = Tucker–Lewis index; RMSEA = root mean square error of approximation; SRMR = standardized root mean residual; SES = socioeconomic status. Model-1: a model where the regression coefficient (β) was freely estimated across groups. Model-2: a model where the regression coefficient (β) was constrained to be equal across groups.

**Table 2:** Standardized beta coefficients of the unconstrained models.

Model	Positive Affect	Negative Affect
ASE × low SES interaction	0.35 ***	−0.15 ***
ASE × medium SES interaction	0.34 ***	−0.18 ***
ASE × high SES interaction	0.32 ***	−0.18 ***
ASE × native adolescents interaction	0.33 ***	−0.17 ***
ASE × 1st-generation immigrant adolescents interaction	0.42 ***	−0.13 ***
ASE × 2nd-generation immigrant adolescents interaction	0.37 ***	−0.13 ***

***Note***. *** *p* < 0.001.

## Data Availability

The data that corroborates the findings of the present study are available at: https://invalsi-serviziostatistico.cineca.it (accessed on 1 December 2024).
